# Editorial: Mitochondrial Genomes and Mitochondrion Related Gene Insights to Fungal Evolution

**DOI:** 10.3389/fmicb.2022.897981

**Published:** 2022-04-11

**Authors:** Vassili N. Kouvelis, Georg Hausner

**Affiliations:** ^1^Division of Genetics and Biotechnology, Department of Biology, School of Science, National and Kapodistrian University of Athens, Athens, Greece; ^2^Department of Microbiology, University of Manitoba, Winnipeg, MB, Canada

**Keywords:** fungi, organellar genomes, mitochondrial-nuclear interactions, mobile introns, mitogenomes

Mitochondria are organelles of eukaryotic cells that provide the platform for efficient energy metabolism, Fe/S-cluster biosynthesis, amino acid metabolism, and moreover, they have been linked to apoptosis, senescence, virulence, and drug resistance (Olson, 2001; Osiewacz et al., 2010; Chatre and Ricchetti, 2014; Giordano et al., 2018; Medina et al., 2020).

They originated from an ancestral α-proteobacterial endosymbiont (Margulis, 1970). Several complementary and alternative hypotheses to this firstly endosymbiotic theory have been proposed (for review see Martin et al., 2015 and references therein), which usually indicate with modifications the endosymbiosis of an α-proteobacterium within an archaeon. However, the additional participation of lysogenic viruses to the archaeal progenitor (Bell, 2009) or phage-like infected α-protebacterial progenitors (Varassas and Kouvelis) may have also contributed to the genesis of the proto-eukaryote. Mitochondria are semi-autonomous organelles, since they carry their own mitochondrial (mt) genomes and the components for protein synthesis. However, mitogenomes do not encode for all molecules necessary for the function and structure of this organelle. Maintenance of the mitogenome requires nuclear encoded factors that drive DNA replication, repair and transmission (Freel et al., 2015). Expression of mitochondrial genes is assumed to be regulated at the post-transcriptional level requiring nuclear encoded general and gene specific factors that guide transcription, RNA processing, intron splicing, RNA stability and translation (Lipinski et al., 2010; Varassas and Kouvelis). Mitogenome expression is linked with nuclear gene expression, establishing extensive inter-compartmental crosstalk that can integrate organellar gene expression into the cellular context as influenced by physiological, developmental, and environmental cues. A limited number of studies have shown mitonuclear interactions and more specifically, nuclear mitochondrial compatibility and co-adaptation, probably, are key components in fungal evolution and adaptation (Giordano et al., 2018; Steensels et al., 2021). Recently, Clergeot and Olson showed a link of nuclear and mitochondrial loci that affect radial growth of *Heterobasidion parviporum* heterokaryons (agent of root rot and butt rot of conifers); the mt involved gene has been identified as a unidentified ORF (uORF) (Himmelstrand et al., 2014), correlated to mt plasmids integrated to the mt genome (Medina et al., 2020). Mitogenomes probably encode uORFs and, by definition, these have no known function and homologs. These genes potentially evolved by endogenous events and although these might be viewed as accessory elements (or not essential), uORFs may have lineage specific functions that allow for fungi to adapt to certain environmental conditions or act as key drivers of evolution for host-pathogen interactions (Monteiro-Vitorello et al., 1995; Inoue et al., 2002; Patkar et al., 2012; van de Vossenberg et al., 2018).

In general, fungal mitogenomes contain genes which encode products (RNAs or proteins) involved in translation (the small and large ribosomal subunit RNAs (*rns* and *rnl*) and a set of tRNAs), plus genes encoding protein components involved in the electron transport chain and oxidative phosphorylation. This includes parts of Complex I (subunits of NADH dehydrogenase: *nad*1 to *nad*6 and *nad*4L; except for members of the Taphrinomycota and Saccharomycetaceae and Saccharomycodaceae families of the Saccharomycetales), components of Complex III (*cob*) and Complex IV (*cox*1, *cox*2, and *cox*3), plus members of Complex V (ATP synthase components: *atp*6, *atp*8, and usually *atp*9) (Zardoya, 2020). Mitogenomes can also encode a ribosomal protein (*rps3* or *var1*) and the RNA (*rnp*B gene) component for RNaseP (Lang, 2018). The above-mentioned genes are designated as the conserved elements of fungal mitogenomes, even though it was recently shown that one or more of these genes may be also absent, arbitrarily in fungi independent to their taxonomic position (Korovesi et al., 2018; Fonseca et al.). Mitogenomes also include many accessory genes and elements in their content, besides the uORFs mentioned above. For example self-splicing introns, intron encoded ORFs, uORFs and in some members of the Ascomycota mitochondrial ORFs have been detected that appear to encode putative N-acetyltransferases and amino-transferases (Wai et al., 2019). Variability in mitogenome size is in part due to intergenic spacers, duplications, proliferation of repeats, and insertions of plasmid components or other elements (Bullerwell and Lang, 2005; Himmelstrand et al., 2014; Medina et al., 2020) (Fonseca et al.; Hao). All the above elements render fungal mitogenomes greatly diverse in content and ranging in size from 12.055 to > 500 kb (James et al., 2013; Liu et al., 2020).

Mt protein and rRNA coding genes are, usually, interrupted by introns that based on the RNA secondary structure and their splicing mechanisms can be assigned to either group I or group II introns (Michel and Westhof, 1990; Lang et al., 2007; Prince et al.). Mitochondrial introns are potentially self-splicing but to achieve splicing competent configurations they need to recruit protein factors (reviewed in Prince et al.). Organellar introns can be mobile elements as they encode intron-encoded proteins (IEPs) that may catalyze the movement of an intron from an intron-containing allele to cognate alleles that lack introns, a process referred to as intron homing or retro-homing, if mediated by reverse transcriptase activity (Belfort et al., 2002). Mobile introns (and their ORFs) are often referred to as diversity generating elements and they can be the major sources of mitogenome size polymorphisms within a species (Li et al.; Valenti et al.; Yildiz and Ozkilinc). However, there are examples where size variation and expansion are linked to repeats and not introns (Hao). In some fungal lineages, expansion of the mitochondrial genome is linked to the expansion of intron numbers (Megarioti and Kouvelis, 2020; Mukhopadhyay and Hausner, 2021), offering a possibility of fine tuning mitochondrial gene expression by nuclear factors that are involved in the splicing of group I and II introns (Rudan et al., 2018; Mukhopadhyay and Hausner, 2021; Lin et al.; Yildiz and Ozkilinic).

Mt accessory elements, like intergenic regions, where promoters, GC-clusters and other repetitive elements are located, show greater diversity and evolve faster, compared to the mt coding genes, which remain under purifying selection (Raffaele and Kamoun, 2012; Kolondra et al., 2015; Li et al.; Yildiz and Ozlilinc). Accessory elements can contribute to mt gene shuffling and the variable mitogenome reorganization through promoting recombinational events (Zhang et al.; Hao). This makes comparative mitogenome analyses essential in deciphering their evolution and diversity. In addition, this comparative analysis has been valuable in resolving issues related to fungal taxonomy, population genetics and diagnostics. On a global scale, fungal mitogenomes might be too variable to provide resolution to address some of the deeper phylogenetic issues within the Mycota (Fonseca et al.). As mentioned above, mitogenome architecture (gene composition and synteny) is highly variable among the fungi due to recombination events. These events are promoted by potential hyphal fusion associated with the existence of potential heteroplasmy (Zhang et al.). Combined with repeats promoting intrachromosomal recombination events and the potential horizontal movements of mobile elements (GC clusters, group I and II introns, homing endonuclease genes) plus uniparental inheritance, phylogenies based on mitogenomes have to be interpreted with caution when trying to address deeper phylogenetic questions (Aguileta et al., 2014; Stoddard, 2014; Repar and Warnecke, 2017; Mayers et al., 2021; Fonseca et al.; Hao). With regards to fungal pathogens, mitogenomic approaches have established potential links with fungicide/drug resistance and mitogenome features that can be linked to adaptation to specific hosts (Cinget and Bélanger, 2020; Wai and Hausner, 2021). On the latter issue, Lin et al. observed that among *Rhizoctonia solani* anastomosis groups there was some correlation between mitogenome gene expression patterns and the plant host, offering potential insights into fungal pathogens that have adapted to different hosts.

This special issue provides a cross section of research highlighting the various aspects of comparative mitogenomics and the potential of mitonuclear interactions on fungal adaptation and evolution. Yet, it also shows the need for more work on this topic, starting from improvements in accurate mitogenome annotations to the application of omics and systems biology approaches in unraveling the complexities of mitonuclear interactions and regulatory processes.

## Author Contributions

All authors drafted the Editorial and made direct and intellectual contributions to the work and approved the final version for publication.

## Funding

This work was funded by Natural Sciences and Engineering Research Council of Canada (NSERC), Discovery Grants Program (RGPIN-2020-05332 to GH).

## Conflict of Interest

The authors declare that the research was conducted in the absence of any commercial or financial relationships that could be construed as a potential conflict of interest.

## Publisher's Note

All claims expressed in this article are solely those of the authors and do not necessarily represent those of their affiliated organizations, or those of the publisher, the editors and the reviewers. Any product that may be evaluated in this article, or claim that may be made by its manufacturer, is not guaranteed or endorsed by the publisher.
